# AI-aided detection of malignant lesions in mammography screening – evaluation of a program in clinical practice

**DOI:** 10.1259/bjro.20200063

**Published:** 2021-02-03

**Authors:** Greta Johansson, Caroline Olsson, Frida Smith, Maria Edegran, Thomas Björk-Eriksson

**Affiliations:** 1 Department of Radiology, NU Hospital Group, Trollhättan/Uddevalla, Region Västra Götaland, Sweden; 2 Regional Cancer Centre West, Gothenburg, Region Västra Götaland, Sweden; 3 Department of Radiation Physics, Sahlgrenska Academy, University of Gothenburg, Gothenburg, Sweden; 4 Department of Technology Management and Economics, Centre for Healthcare Improvement, Chalmers University of Technology, Gothenburg, Sweden; 5 Department of Mammography, NU Hospital Group, Uddevalla, Region Västra Götaland, Sweden; 6 Department of Oncology, Institute of Clinical Sciences, Sahlgrenska Academy, University of Gothenburg, Gothenburg, Sweden

## Abstract

**Objectives:**

Evaluation of the degree of concordance between an artificial intelligence (AI) program and radiologists in assessing malignant lesions in screening mammograms.

**Methods:**

The study population consisted of all consecutive cases of screening-detected histopathologically confirmed breast cancer in females who had undergone mammography at the NU Hospital Group (Region Västra Götaland, Sweden) in 2018 to 2019. Data were retrospectively collected from the AI program (lesion risk score in percent and overall malignancy risk score ranging from 1 to 10) and from medical records (independent assessments by two radiologists). Ethical approval was obtained.

**Results:**

Altogether, 120 females with screening-detected histopathologically confirmed breast cancer were included in this study. The AI program assigned the highest overall malignancy risk score 10 to 86% of the mammograms. Five cases (4%) were assigned an overall malignancy risk score ≤5. Lack of consensus between the two radiologists involved in the initial assessment was associated with lower overall malignancy risk scores (*p* = 0,002).

**Conclusion:**

The AI program detected a majority of the cancerous lesions in the mammograms. The investigated version of the program has, however, limited use as an aid for radiologists, due to the pre-calibrated risk distribution and its tendency to miss the same lesions as the radiologists. A potential future use for the program, aimed at reducing radiologists’ workload, might be to preselect and exclude low-risk mammograms. Although, depending on cut-off score, a small percentage of the malignant lesions can be missed using this procedure, which thus requires a thorough risk–benefit analysis.

**Advances in knowledge:**

This study conducts an independent evaluation of an AI program’s detection capacity under screening-like conditions which has not previously been done for this program.

## Introduction

Computer-aided detection (CAD), part of the artificial intelligence (AI) concept, has been applied to mammography screening for the last two decades.^1,2^ Increased recall rates and insufficient improvement in cancer detection have been limiting for clinical use of CAD programs.^1^ More recently, AI algorithms based on deep learning, a complex technique which resembles the mechanism of neural networks in the human brain, have been introduced and show promising results in detecting malignant lesions.^1–3^ However, the algorithms are typically developed using data sets with a large proportion of cancers compared to a screening population.^3^ Additionally, external validation of the programs as well as prospective studies to evaluate their performance in a mammography screening context are widely lacking.^1–3^

Transpara^TM^ (v. 1.4.0, ScreenPoint Medical, Nijmegen, Netherlands), a CAD/AI program for mammography, has recently been available at the mammography department at the NU Hospital Group (Region Västra Götaland, Sweden). The detection capacity of the Transpara program has been reported to be comparable to that of radiologists in several studies.^4–6^ If simultaneously used to aid radiologist’ assessments, the program increases the detection capacity of the radiologist.^4^ However, cancer rates in the studied cohorts have been high, 25–42%, compared to the expected prevalence of 0.5–0.8% in a population screened for breast cancer.^3^ Moreover, one of the studies showed that radiologists perform better than the program when the number of false-positive cases was increased.^5^ Few studies undertake an independent investigation of this and similar program’s detection capacity under real screening-like conditions.^1–3^

This study was conducted with the aim of evaluating the degree of concordance between the Transpara program and radiologists in assessing malignant lesions in screening mammograms.

## Methods and materials

A retrospective study design was used, which included consecutive cases of screening-detected histopathologically confirmed breast cancer in females. The females had undergone screening mammography at the NU Hospital Group (Region Västra Götaland, Sweden) during the period of April 23, 2018–March 3, 2019. Cases of interval cancer were not possible to include since the study period encompassed one round of screening only. Two criteria for exclusion were deployed. Firstly, for females with bilateral breast cancers, one side was excluded in order to perform analyses on a patient/examination level. Secondly, cases missing data on the overall malignancy risk score were excluded, which was due to technical unavailability (Table 1). Having previous history of breast cancer was not an exclusion criterion. The study was approved by the Swedish Ethical Review Authority which waived the need for individual informed consent.

**Table 1.** Characteristics of the study population and distribution of cancer type, the AI program’s delineation of lesions, lesion risk scores and radiologists’ assessments

Number	*n* = **120**		Excluded *n* = **39**
*Age*, years			
Mean ± SD	62 ± 9		63 ± 9
Median (range)	65 (44–74)		66 (44–74)
*Cancer type^a^/ ^b^*	IDC	ILC	IDC / ILC
Number (%)	84 (70)	13 (11)	29 (74) / 2 (5)
*Lesion delineation^c^*	Yes	No	
Number (%)	100 (83)	20 (17)	
Overall malignancy risk score			
Mean ± SD	9,96 ± 0,32	7,35 ± 2,48	
Median (range)	10 (7–10)	8 (3–10)	
*Lesion risk score*	CC	MLO	
Mean ± SD	74 ± 23	66 ± 24	
Median (range)	85 (27–95)	71 (25–95)	
*Radiologists’ assessments^d^*	Detected	Missed	Detected / Missed
Number (%)	105 (88)	15 (13)	33 (85) / 6 (15)
Overall malignancy risk score			
Mean ± SD	9,68 ± 1,22	8,47 ± 2,17	
Median (range)	10 (3–10)	10 (3–10)	

AI, artificial intelligence.

a IDC = invasive ductal cancer, ILC = invasive lobular cancer. Other cancer types: invasive tubular cancer (n = 3; 3%), invasive mucinous cancer (n = 1; 1%), invasive papillary cancer (n = 1; 1%), metastasis of non-breast primary cancer (n = 1; 1%) and unknown type (in situ n = 14; invasive n = 3; total n = 17, 14%).

b Distribution of cancer types in the excluded group, in addition to IDC and ILC: invasive tubular cancer (n = 1; 3%), papillary cancer (n = 2; 5%) and unknown type (n = 5; 13%).

c CC = cranio-caudal projection, MLO = medio-lateral-oblique projection. Missing data: 65 (54%) for CC and 62 (52%) for MLO.

d Cancerous lesion detected by both radiologists versusvs missed by one radiologist.

All data were obtained from medical records at the Mammography Department at the NU Hospital Group (Region Västra Götaland, Sweden) and from the Transpara program. Data were collected after mammography had been carried out and assessed both by radiologists and the program. The radiologists’ independent assessments, the female’s age and the pathoanatomical diagnosis were obtained from the mammography records. The overall malignancy risk score, delineation of the lesion and, when available, lesion risk score for the respective mammographic projection were obtained from the program. Lesion delineation by the program was regarded as successful when the program had visually marked the histopathologically confirmed lesion.

### Mammography screening

Mammography screening at the NU Hospital Group (Region Västra Götaland, Sweden) includes two image projections of each breast, one craniocaudal (CC) and one mediolateral-oblique (MLO) view. Mammograms are independently assessed by two radiologists, who categorise the mammography findings as either ”normal” or ”suspected malignancy”. In the next step, two radiologists (who might have participated in the first assessment) reassess mammograms labelled as “suspected malignancy” and determine cases requiring further investigation. These females undergo additional X-ray imaging and ultrasound as well as fine needle aspiration cytology and/or 14-Gauge core needle biopsy. In this study, the breast cancer diagnosis was based on post-operative histopathological analysis of surgically removed tissue and/or core needle biopsy in all cases.

### The Transpara program

**Figure 1.**
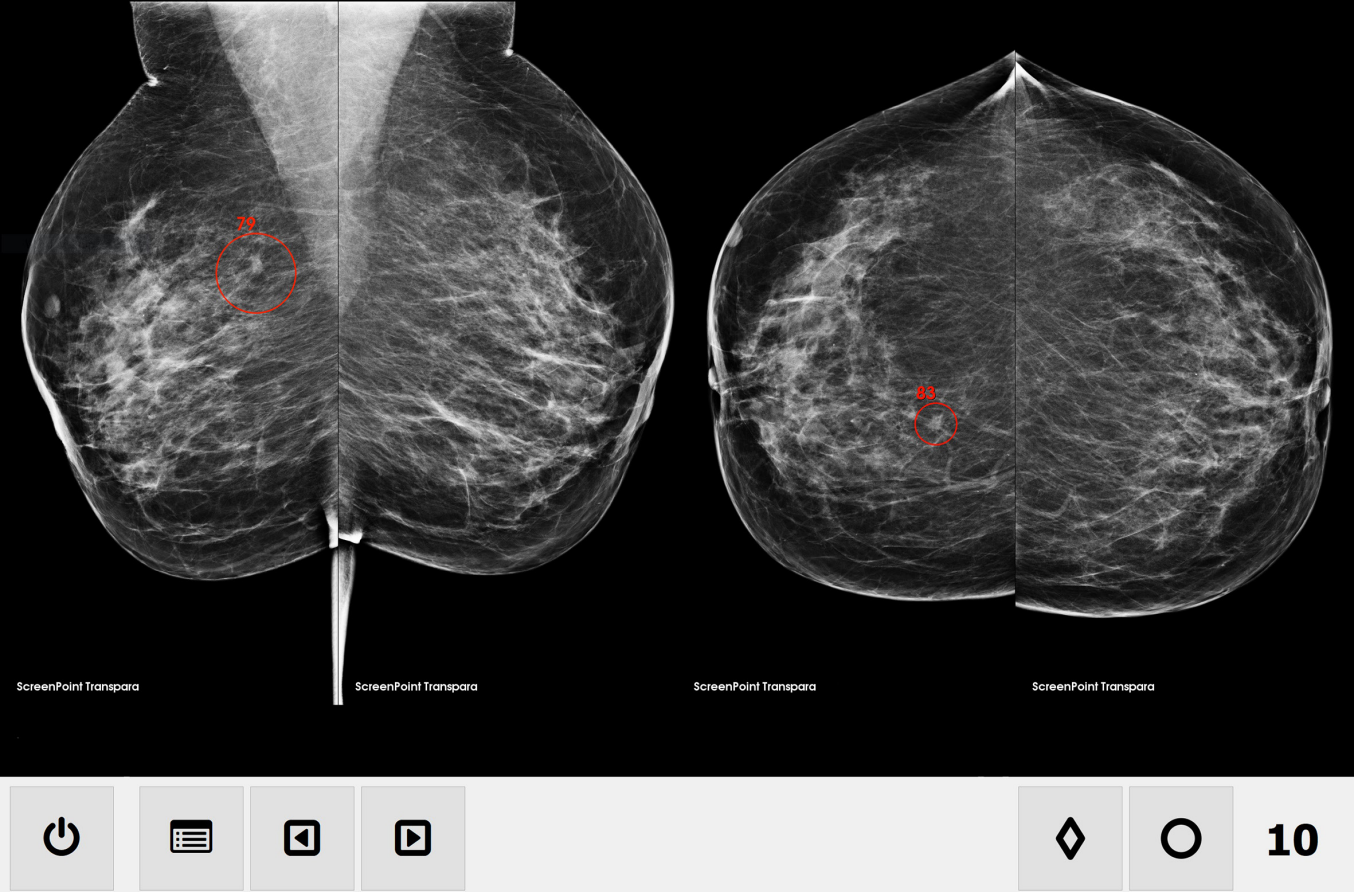
Transpara mammogram overview, MLO (left) projection and CC (right) projection. The overall malignancy risk score (in this case, 10) is indicated in the lower right-hand corner. Lesions are delineated with a red circle and assigned a risk score. In this case, a lesion in the right breast is given a risk score of 79% in the MLO image projection and 83% in the CC image projection. Published with permission of ScreenPoint Medical. CC, craniocaudal; MLO, mediolateral-oblique.

Transpara is a CAD/AI program based on deep learning, developed to detect malignant lesions in mammograms.^4^ It can be used either to aid manual assessments or to make independent assessments. The program visually delineates lesions in the mammogram and assigns a risk score in percent for each detected lesion (lesion risk score). Moreover, an overall malignancy risk is obtained (Transpara score). The Transpara score ranges from 1 (low risk) to 10 (high risk) and is distributed in a pre-calibrated manner in which the program is designed to categorise mammograms in ten groups of equal size (Figure 1).

After introduction of the Transpara program, assessment of screening mammograms proceeded according to the above-mentioned routine with two independent radiologists, but with the additional possibility of simultaneous use of the program for decision support, *i.e.* the radiologists could use the program for simultaneous decision support and were thus not blinded to the results of the program.

### Statistics

Descriptive statistics are given as mean ± standard deviation, median and range for all variables. Percentages were calculated based on the number of patients in the entire study population, if not otherwise stated. Differences between groups and relationships between variables were analysed with Wilcoxon’s Signed Rank test and regression analyses. A two-sided *p*-value of <0.05 was regarded as indicating statistical significance. Data processing and calculations were carried out in the R programming environment for statistical computing (v. 3.5.3, R Foundation for Statistical Computing, Vienna, Austria).

## Results

### Study population

During the study period, 26,373 females underwent mammography in the screening program at the NU Hospital Group (Region Västra Götaland, Sweden), of whom 159 (0.6%) were diagnosed with histopathologically confirmed breast cancer. Bilateral breast cancer was diagnosed in two females. Technical limitations regarding the program’s availability and server storage capacity resulted in exclusion of 39/159 (25%) cases due to missing data on the overall malignancy risk score. Characteristics of the final study population of 120 females and the excluded cases are shown in Table 1.

### Overall malignancy risk score

**Figure 2.**
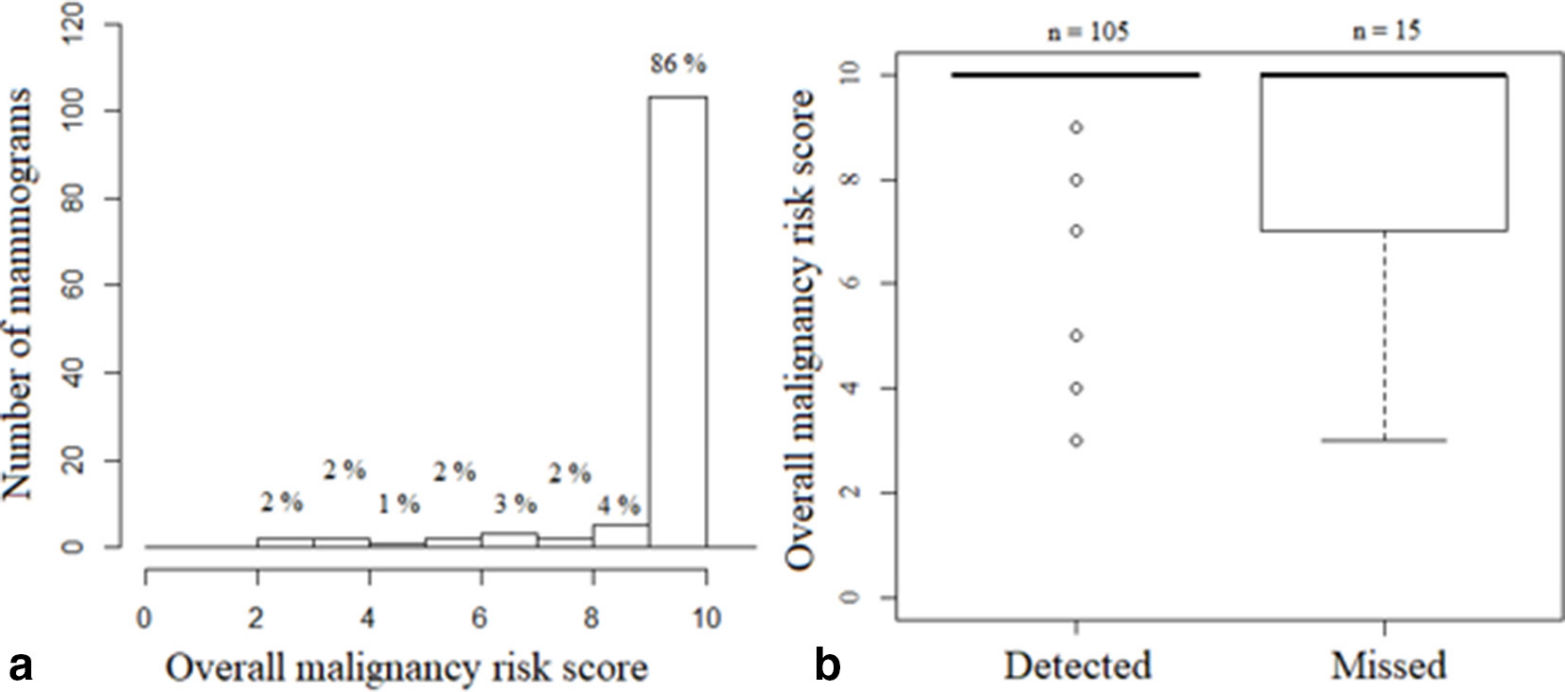
(a) Distribution of the AI program’s overall malignancy risk scores. (b) Overall malignancy risk score related to radiologists’ assessments (cancerous lesion detected by both radiologists versus missed by one radiologist). AI, artificial intelligence.

The program assigned score 10 in 103/120 (86%) mammograms (Figure 2a). The remaining 17 mammograms were assigned: score 9 (*n* = 5), score 8 (*n* = 2), score 7 (*n* = 3), score 6 (*n* = 2), score 5 (*n* = 1), score 4 (*n* = 2), score 3 (*n* = 2).

### Lesion delineation and risk scores

The program successfully delineated the cancerous lesion in 100/120 cases (83%, Table 1). The mean risk score was 74% for CC projections (available for 55/120 [46%] mammograms) and 66% for MLO projections (available for 58/120 [48%] mammograms), a statistically significant difference between the image projections (*p* = 0.02, Table 1).

### Radiologists’ assessments

The cancerous lesion was detected by both radiologists in 105/120 cases (88%, Table 1). The AI program’s overall malignancy risk score was lower in cases where the lesion was missed by one radiologist (15/120, 13%) than in cases where the lesion was detected by both radiologists (8.5 ± 2.2 *vs* 9.7 ± 1.2; *p* = 0.002; Figure 2b). The program was only able to delineate the lesion in 47% of the cases where the lesion was missed by one radiologist, in contrast to cases where the lesion was detected by both radiologists, in which the program delineated the lesion in 89% of the cases.

### Lesion characteristics

The distribution of cancer types in the study population is shown in Table 1. Subgroup analyses for cases assigned an overall malignancy risk score of 10 (*n* = 103) and ≤5 (*n* = 5) showed a similar distribution of cancer types as the study population as a whole: invasive ductal cancer (72% and 80% vs 70%), invasive lobular cancer (11% and 0% vs 11%) and other cancer types (17% and 20% vs 19%).

## Discussion

In this study, we have compared the ability of the AI program Transpara to detect malignancy-suspect lesions with that of radiologists in 120 mammograms with screening-detected histopathologically confirmed breast cancer at the NU Hospital Group (Region Västra Götaland, Sweden) in 2018 to 2019.

We found that the program assigned the highest overall malignancy risk score (10/10) in 86% of the mammograms and that the program successfully delineated the lesion in 83% of the cases. Consequently, 14% of the cancer cases were assigned lower scores than 10, although not the lowest scores (1–2) and the lesion was not delineated in 17% of cases. Previous studies have found that Transpara detects malignancy-suspect lesions to an extent comparable to that of radiologists, measured as area under the receiver operating characteristic curve, as sensitivity and specificity or as true positive fractions.^4–6^ It is, therefore, reasonable to expect that most cancers in our study would be assigned an overall malignancy risk score of 10. However, that did not correspond with our findings and can suggest deficiencies in the program’s performance when applied to a real screening population. We also found that the same lesion often was assigned a higher lesion risk score in the CC projection than in the MLO projection. This indicates that the program has some built-in systematic differences with respect to image projection. Since AI-based programs are calibrated for the material they have been trained on, clinical usefulness will be impaired if a limited or non-representative case selection is used in its construction or validation process.^1,3^

There is a general need for external validation of AI programs in large representative screening populations.^3^ Three commercially available AI algorithms have recently been subject to an external, independent evaluation in a case–control setting.^7^ One of the studied algorithms showed a significantly higher performance compared with the other two algorithms, highlighting the importance of construction and training data for external validity. Interestingly, combining the best performing AI with the first reader/radiologist was more successful than combining the first and second readers regarding cancer detection rate, however, at the cost of increasing false-positive cases.^7^ Another study, also evaluating a different AI program than Transpara, illustrates the complexity both in calibrating and in evaluating AI algorithms based on retrospective data.^8^ For instance, reasonable cut-off levels must be chosen for the AI assessments (that are provided on a continuous scale) to enable comparison with the human assessments (that are provided on a binary scale: negative/suspected malignancy). This study also provides evidence for the potential complementary roles played by AI systems and radiologists, as both are expected to miss a small proportion of cancers, but not necessarily of the same type. However, this study, as well as many other AI evaluations, are limited by the retrospective setting and the lack of follow-up/biopsy-verification of mammograms with AI-detected lesions which were classified as “normal” by radiologists.^3,9^ This impairs the possibility for evaluation of AI-detected lesions and makes it difficult to determine the amount of actual AI-detected cancers versus false-positive cases.^9^

Another important finding in our study was that lesions missed by radiologists at single-reading were associated with both lower overall malignancy risk scores and with lesion delineation to a lower extent. This is important when discussing the possibility for stand-alone assessments by this and similar programs to reduce workload for radiologists. It has been suggested that Transpara could be used to exclude low-risk cases from the radiologist’s assessment.^10^ If a cut-off score of 5 (overall malignancy risk score) is applied, scores ≤5 by the program will be regarded as “normal”. The radiologists’ workload would then decrease by 47% while the rate of missed cancer cases would reach 7%.^10^ In our material, there were five cancer cases (4%) with scores ≤5, which would have been missed if relying on the program alone (N. B. one of these five cases was also missed by one of the radiologists). Applying an alternative cut-off score of 2 was discussed in the same study,^10^ which would lead to less decrease in workload (17%) but fewer missed cancer cases (1%). Reducing radiologists’ workload by excluding low-risk cases was also evaluated in a retrospective simulation study concerning another AI program.^11^ This study concluded that using sole AI assessments in 60–80% of the cases would result in only a proportion of ≤2.6% missed cancers with a substantial workload reduction.^11^ Moreover, the study showed a potential increase in cancer detection (12% *vs* 27%) if 1% *vs* 5% of females with the highest AI risk scores were selected to an enhanced assessment group, which for instance would include supplemental imaging with magnetic resonance tomography.^11^ In comparison, the Transpara program includes 10% of all mammograms in the top-risk category, potentially leading to a large number of cases selected for radiologists’ assessment or recalls if it is used alone in a real screening population.

With account to all above-mentioned aspects, remembering that all of the histopathologically confirmed cancerous lesions in our data had an overall malignancy risk score ≥3, a safe alternative (given that this can be confirmed in future studies) would be to only trust the scores for the low-risk group, *i.e.* cases with a score ≤2. By excluding normal cases, the need for manual double-reading would thereby be reduced.

### Strengths and limitations

The main strength of this study is the investigation of cases with screening-detected histopathologically confirmed breast cancer in a large unselected consecutively screened population. The main limitation of this study is the case-only setting which prevents evaluation of false-positive cases and assessment of sensitivity and specificity. The case-only design also risks biasing the study in favour of radiologists. Other limitations include the relatively small study population which resulted from limitations in the program’s availability and server storage capacity and a risk of data collection bias. It cannot fully be ruled out that the radiologists were influenced by the program’s results when making their own assessments. Another minor limitation is not to have included the program’s risk score of any other lesions (in addition to the verified cancers).

## Conclusion

AI is increasingly utilised in radiology and has now also been available for mammography screening at the NU Hospital Group (Region Västra Götaland, Sweden). Transpara, an AI-based CAD program, detected the majority of cancerous lesions in the mammograms, also assigning high malignancy risk scores. However, the program in the investigated version is of limited use as an aid for radiologists, due to the pre-calibrated risk distribution and its tendency to miss the same lesions as the radiologists. Moreover, our findings indicated an unexpected discrepancy in lesion risk scores between the two studied image projections, which suggests that the program suffers from a systematic built-in difference with respect to image projection. A potential future use for the program, aimed at reducing radiologists’ workload, might be to preselect and exclude low-risk mammograms from radiologists’ assessment. Although, depending on cut-off score, a small percentage of the malignant lesions can be missed using this procedure, which thus requires a thorough risk–benefit analysis.
